# Evaluating the access of slum residents to healthcare centers in Kermanshah Metropolis, Iran (1996–2016): A spatial justice analysis

**DOI:** 10.1016/j.heliyon.2022.e12731

**Published:** 2022-12-30

**Authors:** Alireza Zanganeh, Arash Ziapour, Reyhane Naderlou, Raziyeh Teimouri, Parisa Janjani, Komali Yenneti

**Affiliations:** a Social Development and Health Promotion Research Center, Health Institute, Kermanshah University of Medical Sciences, Kermanshah, Iran; b Cardiovascular Research Center, Health Institute, Imam-Ali Hospital, Kermanshah University of Medical Sciences, Kermanshah, Iran; c University of Zanjan, Zanjan, Iran; d UniSA Creative, University of South Australia, Adelaide, Australia; e School of Architecture and the Built Environment, University of Wolverhampton, UK

**Keywords:** Slum, Healthcare, Inequity, Spatial analysis

## Abstract

**Background:**

Proper access to health care centres and services is one of the key indicators of health justice, and it is more than ever important in slums.

**Objective:**

This aim of this research is to evaluate the accessibility of health care centres to slum residents in the Kermanshah metropolis, Iran during the period 1996–2016.

**Methods:**

In this cross-sectional study, data was obtained from the Census of Iran for the periods 1996, 2006 and 2016. Information on the number and location of health care centres was collected from the Kermanshah University of Medical Sciences. Network Analysis modelling method in Arc/GIS10.6 software was used to evaluate the accessibility of people to health centres.

**Results:**

The results show that the spatial pattern of health centres in Kermanshah was random during 1996, 2006 and 2016, but the spatial pattern of poverty in the metropolis was clustered. In addition, the distribution of health centres was not consistent with the population densities. However, the overall population with inappropriate access to health centres in the slums of Kermanshah metropolis decreased over the study period (1996–54.02%, 2006–51.09%, and 2016–34.71%).

**Conclusions:**

The findings of the study reveal that access to health care services by the slum population is not consistent with the increase of health care centres. This means that health policymakers were unsuccessful to provide the required health care services for the slums.

## 1 Introduction

Spatial justice, referred to as “justice in physical space”, emphasises a just distribution of available resources within and across geographical spaces [1, 2]. It involves respecting the fundamental human rights of all people in a society, and promoting inclusive spatial development in order to reduce economic inequalities and social polarisation [1]. More specifically, the pursuit of spatial justice can be achieved if the organisation of space and the allocation of resources follow the principles of equity and respect for human rights [1, 3, 4, 5, 6]. Balanced spatial distribution of resources is one of the most important indicators of social justice, although spatial justice is not a substitution for social justice [1, 3]. The emphasis is on redressing the exclusion and advancing the inclusion of poverty-stricken and low-income groups in spatial development processes [1, 7, 8].

From a spatial justice perspective, health justice encompasses the proportional distribution of health-efforts and -services and access to health service delivery centres without discrimination and disparities among urban residents [9]. A critical issue in health justice — as a social justice strategy— is how health services are distributed in urban areas. Health injustices include not only inequalities in the determinants of health, but also access to resources needed to improve and maintain health or health outcomes [10, 11]. Health justice is important because health is a basic human right and its realisation will eliminate inequalities resulting from differences in health conditions, such as disease or disability, and provide opportunities to enjoy and pursue everyday life. A common characteristic among different groups—poor or marginalised persons, racial and ethnic minorities, and women—that experience health injustices is lack of political, social or economic power [10]. Spatial imbalances and uneven distribution of health services and facilities are more pronounced in developing countries [12, 13].

Previous studies on public health in the Middle East have focused on threats such as climate change, war, inadequate nutrition, gender inequality, rapid urbanisation and socio-economic inequalities [14, 15]. Few studies have explored the role of deprivation and access to health care in the outcomes of public health. A small body of scholarship suggest that the relationship between neighbourhood economic characteristics and access to community resources should be commensurate [16, 17]. Even scantier is the literature on the relationship between access to health care services and health outcomes in slums. Attention to this issue is significant as the majority (60–80%) of urban slum residents in developing countries, such as Iran, live in informal settlements characterised by penurious sanitation and hygiene, overcrowding and poor housing, and unfavourable public healthcare services [18, 19]. The burden of disease and illness in urban informal settlements tends to be high due to poor coverage of effective preventive and therapeutic interventions [20, 21, 22, 23]. The near absence of basic public amenities in slums often leads to the mushrooming of several small substandard clinics that serve large slum populations but fail to provide integrated primary healthcare [24]. While private health care providers attempt to bridge the gap in demand for primary healthcare services in slums, they lack the capacity and cannot guarantee service quality due to their profit-centric nature [25]. All these issues have negative implications on access to healthcare services among slum residents [26]. Despite recent efforts to expand universal health coverage, more evidence is needed on barriers and drivers of access to healthcare services in order to improve coverage and performance of primary healthcare systems [27].

In this context, the aim of this study is to evaluate the accessibility of health care services to slum residents in the Kermanshah metropolis, Iran during the period 1996–2016. This study considers the population with and without access to health care centres based on two genders and three age groups [0–14 years (children), 15–64 years (adults) and over 65 years (elderly)]. Kermanshah metropolis is on the path of urban-rural migration. This has caused many social, cultural, and economic challenges including poverty, unemployment, high divorce rates, suicidal ideation and attempted suicides [28, 29], self-immolation, and high rates of HIV/AIDS, most of which are encountered more frequently in poor and marginalised areas. The lack of appropriate access to health services only add to these problems [30, 31, 32, 33]. As such, Kermanshah is a fertile case study to asses the accessibility of health care services to slum residents.

The findings of this research can a) help urban planners, health researchers, and policymakers to identify underserved areas and to improve accessibility of health services to vulnerable population; and b) provide a better understanding of the complex relationship between the spatial distribution of health care centres and slums. Awareness on access to healthcare services and associated factors in resource-poor urban settings may help in planning and implementing interventions on health care services.

## 2 Methodology

### 2.1 Design

In this cross-sectional study, the Iranian censuses of 1996, 2006 and 2016 were used. In addition, data for each neighbourhood were also separately used. The spatial patterns of poverty variables were evaluated separately for each neighbourhood. To assess the urban poverty across the statistical blocks, a combination of economic indexes (dependency burden, unemployment rate, male unemployment, general activity rate, employment ratio, gross dependency burden, dependency ratio, employment rate, net dependency burden, female unemployment, economic participation, women's participation in economic activities, overhead rate, livelihood burden, population economic burden), social indexes (youth population, aging rate, average household size), cultural indexes (literacy rate, illiteracy rate, illiteracy rate among, literacy rate among adults) and structural indices (population density, density of residential units, population density in residential units, gross density of residential units, the net density of residential units, household density in residential units, individual density in residential units, residential density of population, per capita net housing, per capita gross housing) [33, 34, 35] were considered and then classified into three groups of lower class, middle class and upper class. Information about health centres was collected from the Kermanshah University of Medical Sciences.

Existing research shows that many decisions in health care and planning are related to location issues. In this regard, Geographical Information System (GIS) can be a powerful tool for evaluating the spatiality of health and health policies around the world [36, 37]. In addition, GIS has been used for health needs assessment, planning and evaluating health delivery services, spatial analysis of access to health services and accessibility of vulnerable and disadvantaged populations to health care centres [38].

In this study, Network Analysis modelling method in ArcGIS10.6 software was used to evaluate the accessibility of people to health centres. The basic map of Kermanshah city and access roads were used for modelling. The network of passages and health centres were digitised in ArcGIS software, while topology and spatial relationships between passages were created using Arc Catalog. Rules related to the duration of walking were applied using the Network Analyze tool. In transportation technical calculations, a person's normal walking speed is 0.75–1.25 m per second [39]. In this study, the average walking speed was assumed to be 1 m/s. The standard radius for access to health care centers was set at 750 m [17,39]. Considering the defined standard radius and average walking speed of a person, the considered walking time from home to the health centre is 12.5 min. Then, using the extensions of Network Analyze tool, boundaries for the areas of service delivery provided by the health centres – i.e., accessibility of slum residents - were created. Populations with and without access to health care centres were calculated using the intersect and symmetrical difference tools. The populations considered in this study included both male and female genders and three age groups [0–14 years (children), 15–64 years (adults) and over 65 years (elderly)] in slums.

The following models were used to identify spatial patterns of health centres and slums.

The Average Nearest Neighbour ratio is given as:

(1) $$ANN=\frac{{\bar{D}}_{0}}{{\bar{D}}_{E}}$$

In which ${\bar{D}}_{0}$ is the observed mean distance between each feature and its nearest neighbour:

(2) $${\bar{D}}_{0}=\frac{\sum_{i=1}^{n}d_{i}}{n}$$

And ${\bar{D}}_{E}$ is the expected mean distance for the features given in a random pattern:

(3) D‾E=0.5nA

In the above equation, $d_{i}$ equals the distance between the feature i and its nearest neighbouring feature. n corresponds to the total number of features, and A is the area of a minimum enclosing rectangle around all features, or its user-specified value [40, 41, 42].

The average nearest neighbour z-score for the statistic is calculated as:

(4) $$Z=\frac{{\bar{D}}_{0}-{\bar{D}}_{E}}{SE}$$

where

(5) SE=0.26136n2A

**Moran's I:** The Moran's I tool measures spatial autocorrelation based on both feature locations and feature values simultaneously. Given a set of features and an associated attribute, the Moran's I evaluates whether the pattern expressed is clustered, dispersed, or random. The tool calculates the Moran's I Index value, z-score and p-value to evaluate the significance of that Index. P-values are numerical approximations of the area under the curve for a known distribution, limited by the test statistic [40, 41, 42].

The Moran's I statistic for spatial autocorrelation is given as:

$$I=\frac{n}{S_{0}}\frac{\sum_{i=1}^{n}\sum_{j=1}^{n}w_{,ji}z_{i}z_{j}}{\sum_{i=1}z_{i}^{2}}$$

In which $z_{i}$ is the deviation of an attribute for a feature i from its mean, $\left(x_{i}-X\right),w_{i,j}$ is the spatial weight between features i and j,n is equal to the total number of features, and $S_{0}$ is the aggregate of all the spatial weights:

$$S_{0}=\sum_{i=1}^{n}\sum_{j=1}^{n}w_{ji}z_{i}z_{j}$$

The ZI-score for the statistic is computed as [40, 41, 42]:

$$ZI=\frac{I-E\left[I\right]}{\sqrt{V\left[I\right]}}$$

where: E[I]=−1(n−1)

$$V\left[I\right]=E\left[I^{2}\right]-E{\left[I\right]}^{2}$$

**Kernel Density:** The Kernel Density tool calculates the density of population and health centres in a neighbourhood around those features. The Kernel Density is given as:

$$The\;Kernel\;Density=0.9\;\times \;\min\;\left(SD,\;\sqrt{\frac{1}{Ln\left(2\right)}}\times D_{m}\;\right)\times n^{-0.2}$$

where: SD is the standard distance, Dm is the median distance, n is the number of population and health centres [40, 41, 42, 43].

## 3 Results

### 3.1 Average nearest neighbour (NNR) for health centres and poverty during 1996, 2006, and 2016

**Figure 1:**
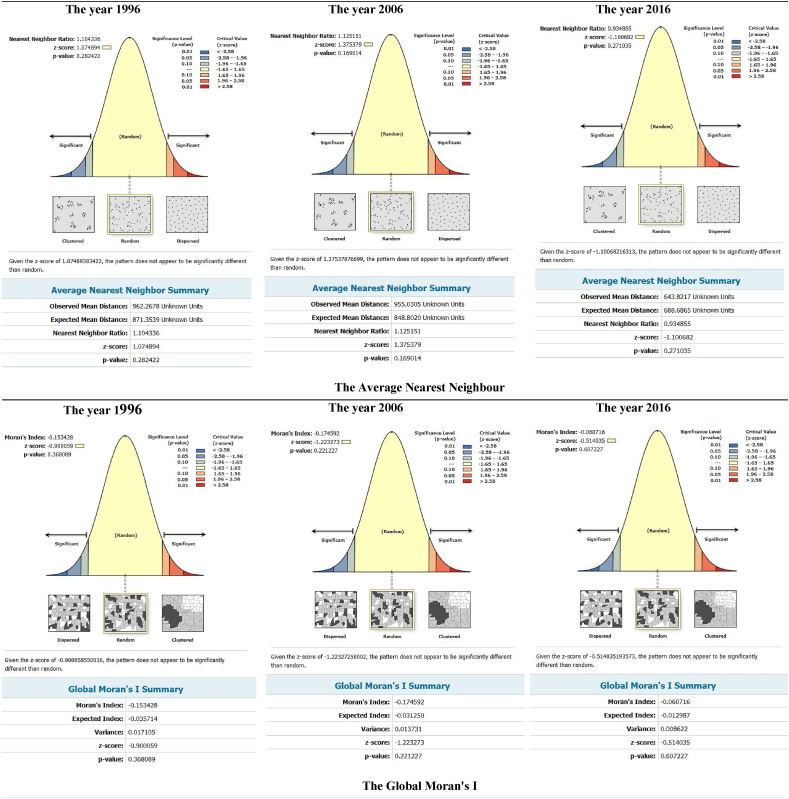
The spatial correlation of health centres (Average Nearest Neighbour and z-score) and the spatial pattern of health centres (Global Moran's I and z-score) in Kermanshah Metropolis during 1996, 2006, and 2016.

The results of the spatial pattern of health centres in Kermanshah show that NNR and z-score in 1996, 2006, and 2016 were (1.10, 1.07), (1.12, 1.37) and (0.93, −1.10), respectively. Moran I index and z-score in 1996, 2006, and 2016 were (−0.15, −0.90), (−0.17, −1.22) and (−0.06, −0.15), respectively. These results indicate that the spatial pattern of health centers in Kermanshah was random, which means that there was no specific spatial planning for the establishment of health centres in Kermanshah (Fig. 1).

**Figure 2:**
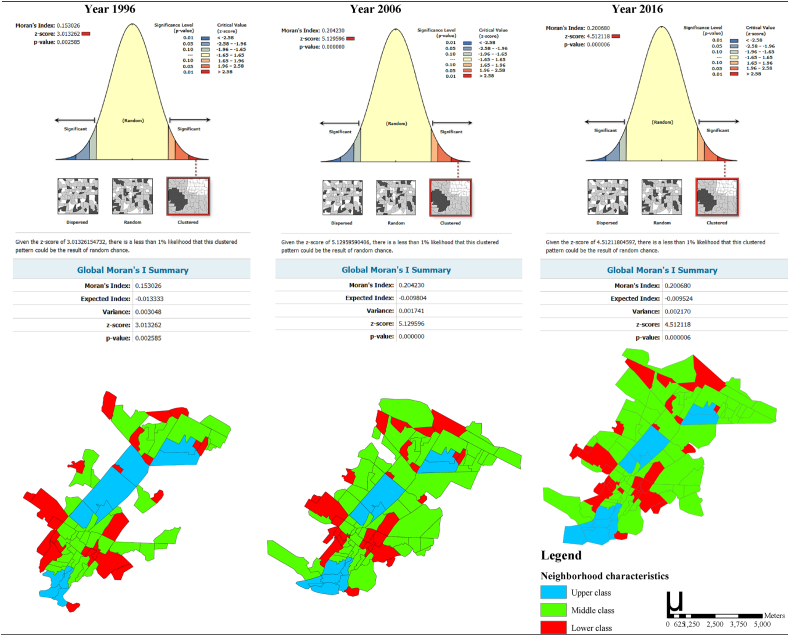
The spatial correlation of poverty (Moran's I index and z-score) in all neighbourhoods (lower class, middle class and upper class) of Kermanshah Metropolis during 1996, 2006, and 2016.

The results of the spatial pattern of poverty in Kermanshah reveal that the Moran I index and z-score in 1996, 2006 and 2016 were (0.15, 3.01), (0.20, 5.15) and (0.20, 4.51), respectively. These findings suggest that the spatial pattern of poverty in Kermanshah metropolis was clustered over the study period (Fig. 2).

### 3.2 Kernel density for health care centre and population in 1996, 2006, and 2016

**Figure 3:**
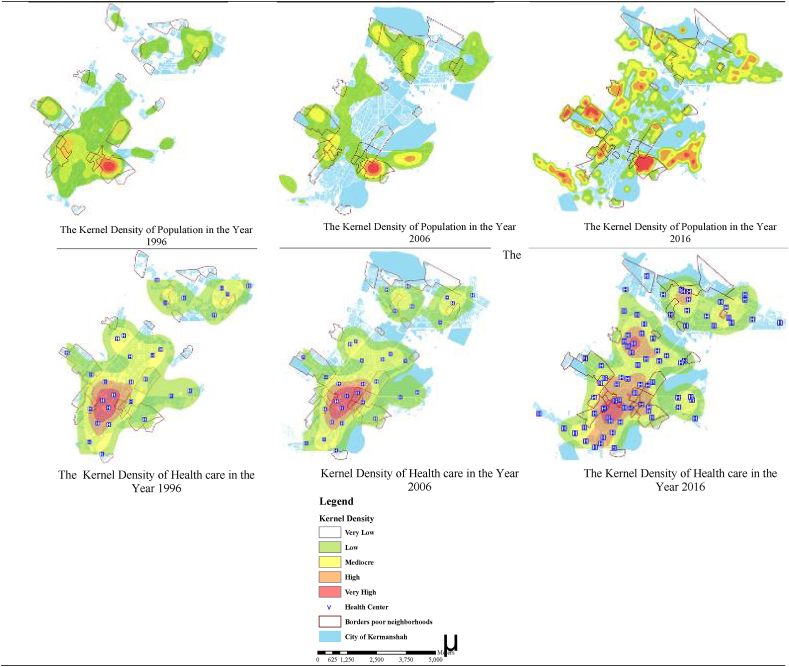
The kernel density estimation test, the highest density of health centers in Kermanshah Metropolis during 1996, 2006, and 2016.

According to the results of the kernel density estimation test, the highest density of health centres in both 1996 and 2006 was observed in the centre of Kermanshah metropolis. In 2016, along with the city centre, high densities of health centres were observed in both north and northwest along with the city centre (Fig. 3).

On the other hand, in 1996, high population densities were observed in the eastern, western and north-eastern regions. In 2006, the population concentration was mainly in the east of Kermanshah, and in 2016, new population centres emerged on the edge of the city. This finding suggests that the distribution of health centres is inconsistent with population density (Fig. 3).

### 3.3 Access to health centers through travel time by walking in 1996, 2006, and 2016

**Figure 4:**
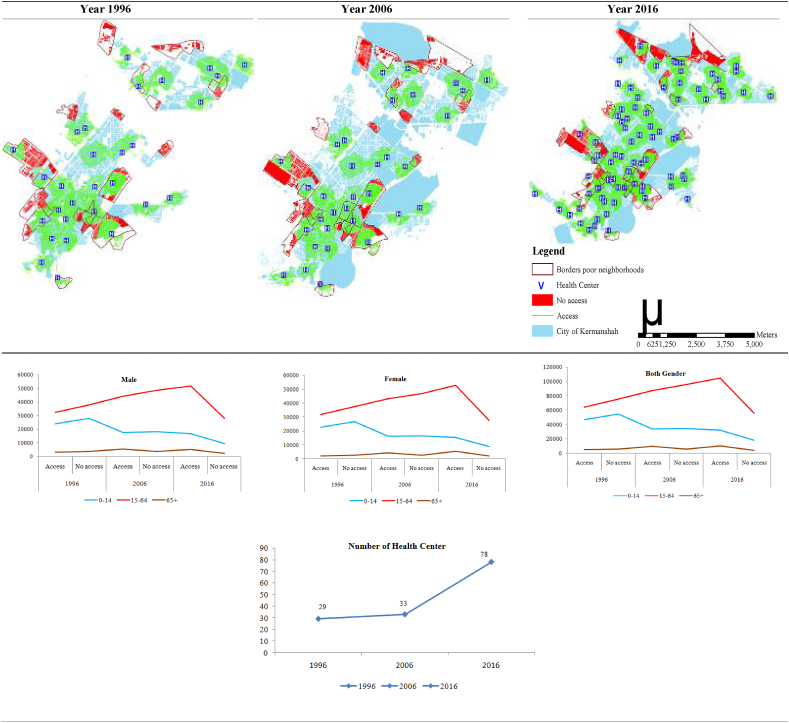
Network analysis model of travel time (by walking) to the nearest health care centre and the population with access/no access in 1996, 2006, and 2016.

The results show that the percentage of population with inappropriate access to health centres in 1996, 2006 and 2016, was 54.02%, 51.09%, and 34.71% respectively. This finding indicates a declining trend in the population without proper access to health centres during the study period. However, this result also suggest that at the end of the study period, 34.71% of the population still did not have appropriate access to health centres. It is important to note that most of this population is in the 0–14 age group (Fig. 4 and Table 1).

**Table 1:** The status of population accessibility to health care centres in Kermanshah Metropolis (1996, 2006, and 2016).

			Male				Female				Total of population			
Year			0–14	15–64	65+	Total	0–14	15–64	65+	Total	0–14	15–64	65+	Total
**1996**	**Access**	N	24014	32529	2940	59483	22755	31727	2312	56794	46769	64256	5252	116277
		%	46.12	46.11	45.57	46.1	45.99	45.78	45.77	45.86	46.05	45.95	45.66	45.98
	**No access**	N	28046	38012	3512	69570	26727	37583	2740	67050	54773	75595	6252	136620
		%	53.88	53.89	54.43	53.9	54.01	54.22	54.23	54.14	53.94	54.05	54.34	54.02
	**Total**	N	52060	70541	6452	129053	49482	69310	5052	123844	101542	139851	11504	252897
		%	100	100	100	100	100	100	100	100	100	100	100	100
**2006**	**Access**	N	17529	44316	5364	67209	16171	43154	4348	63673	33700	87470	9712	130882
		%	49.24	47.57	60.47	48.83	49.32	47.91	61.49	49	49.28	47.74	60.92	48.91
	**No access**	N	18069	48847	3507	70423	16616	46923	2723	66262	34685	95770	6230	136685
		%	50.76	52.43	39.53	51.17	50.68	52.09	38.51	51	50.72	52.26	39.08	51.09
	**Total**	N	35598	93163	8871	137632	32787	90077	7071	129935	68385	183240	15942	267567
		%	100	100	100	100	100	100	100	100	100	100	100	100
**2016**	**Access**	N	16774	51929	4932	73635	15257	52730	5607	73594	32031	104659	10539	147229
		%	64.07	64.91	69.97	65.03	63.38	65.61	71.58	65.55	63.74	65.26	70.82	65.29
	**No access**	N	9408	28074	2117	39599	8817	27634	2226	38677	18225	55708	4343	78276
		%	35.93	35.09	30.03	34.97	36.62	34.39	28.42	34.45	36.26	34.74	29.18	34.71
	**Total**	N	26182	80003	7049	113234	24074	80364	7833	112271	50256	160367	14882	225505
		%	100	100	100	100	100	100	100	100	100	100	100	100

In 1996, 2006 and 2016, the city of Kermanshah had 29, 33, and 78 health centres, respectively. Although the total number of health centres has increased over time, more than one-third of the slum residents did not have appropriate access to health centres at the end of the study period (Fig. 4 and Table 1).

## 4 Discussion

This study, the first of its kind in Iran, has examined slum residents' access to health services in Kermanshah metropolis, Iran during 1996, 2006 and 2016 and by doing so, challenged the health policies in Iran. Several important findings can be drawn from this study. First, the results of this study show that the spatial pattern of health centres in Kermanshah followed random patterns during the study period (1996–2016). This finding is in line with the results from other studies in Iran that have highlighted the country's organic growth, lack of health care planning [44, 45], and weak local management system and policies related to the development of health centres [40]. The random spatial patterns of health centres are the result of poor planning and can lead to increased health inequalities. The disruption of service delivery in cities can also create social inequalities, social crises and complex spatial problems [46]. Access to neighbourhood services and resources can further affect a person's chance of living and their ability to lead a healthy life [14, 16]. On the contrary, dispersed patterns of services signifies the existence of planned and socially just development. In order to achieve a proper distribution of health care centres, future health policies should be aimed at changing the spatial distribution pattern of health care centres from random to dispersed [44].

Second, the spatial pattern of poverty in Kermanshah metropolis in 1996, 2006 and 2016 showed a clustered distribution. Similar findings were observed in Hong Kong, where areas with a high degree of poverty were systematically clustered [47]. This finding is also consistent with studies from large developed Western cities [48, 49]. However, it can be argued that poverty in the West is mainly concentrated in the inner-city areas [50], while the concentration of high poverty levels in Hong Kong and Kermanshah are found in both the inner-city and suburban areas. Other research results also suggest that the formation of poverty occurs in clustered pattern and is an important negative social phenomenon with a spatial dimension that continuous to affect people's lives [33, 51, 52]. Economic factors, class segregation, racial discrimination, population structure, public policies [52], and inaccessibility to health care centres have been found to be major drivers in poverty distribution [47].

Third, Kermanshah city centre had the highest density of health centres during 1996 and 2006. In 2016, the density of health centres stretched north and northwest from the city centre. In 1996, the eastern, western and northeastern areas of the city observed the highest population densities, while in 2006, the eastern part of Kermanshah recorded the highest population densities. In 2016, densely populated centres formed on the fringe areas of Kermanshah metropolis. Migration from rural and other areas of Kermanshah province to Kermanshah metropolis has increased in recent years [53, 54]. The increase in the number of migrants may be due to inappropriate living conditions (including health services) in other parts of the province [40], and better services in the Kermanshah metropolis although further research is required on this topic. A study in India found that people living in slums had better health conditions than those living in rural areas, resulting in residents to prefer living in informal settlements in cities than in rural areas [55]. Similarly, the results of Murad et al. (2018) revealed that large parts of Jeddah City - mainly areas located in the northern districts of the city - have low accessibility to health centres and are considered as un-served populations [56].

Fourth, the results of this study suggest that the distribution of health centres is not consistent with population densities, indicating poor policymaking. This situation can lead to increasingly unstable financing health systems, risky behaviours of people, and unhealthy lifestyles in society [57]. Various other studies have found a range of other challenges to health care access in Iran. For example, Rahimi et al. (2022) mentions barriers to access as one of the challenges to health care performance in Iran [58]. Mehrolhassani et al. (2018) reported that geographical, cultural, and financial factors are key challenges to access to health care services in Iran [59]. Another study found that Iran's health care system has been facing challenges related to governance, human resources, service delivery, technology, financing and information systems. They further argue that these challenges have been weakening Iran's health care system, whose current governance structure is unable to meet new demands, ultimately leading to reduced access to and use of healthcare services [60]. On the other hand, urban policies based on improper planning and disregarding social analysis, city culture and the political economy of space have been leading to widening of inequalities, spatial segregation, marginalisation and unbalanced population development in urban areas [61].

Finally, the results show an overall increase in health centres in Kermanshah metropolis. The total number of health centres increased from 29 in 1996 to 33 in 2006 and 78 in 2016. However, the number of health centres centres in slums in 1996, 2006 and 2016 was 8, 9 and 18 respectively, which indicates that the mean share of poor neighbourhoods was 23.7%. At the same time, the slum population with inappropriate access to health centres in the Kermanshah metropolitan area decreased (1996–54.02%, 2006–51.09%, and 2016–34.71%). It is worth noting that despite the increase in the number of health centres, 34.71% of slum residents still do not have adequate access to health centres, the majority of whom are children aged 0–14. Inaccessibility to health care centers in slums has the potential to increase mortality [62]. Accessibility is a very important factor in the use of health care services in slums, as demonstrated by studies in India [63] and Africa [64].

To summarise, poverty and poor health are often the results of poor investment in public health infrastructure and essential health services Ahmed et al. (2022). Health disadvantages in informal settlements and slum communities in cities are exacerbated by inequities in health service availability, inhibiting the abilities of families to protect themselves from preventable diseases. This locks families and communities into multi-generational cycles of health injustices [65]. This situation reflects inadequate spatial planning in this regard and lack of investment in the health of slum residents.

## 5 Conclusions

The findings of this study provide a better understanding of the complex relationship between the spatial distribution of the health care centres and slums. Given the urbanisation trend of Kermanshah metropolis in recent decades, the slum population will grow rapidly in the coming decades. Although the number of health care centers had increased during the study period, the accessibility of slum population to health care services was not consistent with the increasing number of health care centers. This shows that health policymakers have not been successful in providing slums with access to essential health services. The inappropriate distribution of health care centers is the result of a lack of proper planning, which can lead to increased health inequalities in society.

The novelty of this study lies in finding practical and technical solutions to the problem of access to health care centres for marginal communities in Iran by using spatial modelling methods such as Network Analysis, Average Nearest Neighbour, and Kernel Density. In addition, this research covered two genders and three age groups [0–14 years (children), 15–64 years (adults) and over 65 years (elderly)].

This study focused on the spatial accessibility of services and did not consider non-spatial aspects, such as affordability and acceptability. Further, it did not calculate the spatial accessibility of health care centres through cars or public transport. All of these issues can be considered for future research.

## Author contribution statement

Alireza Zanganeh; Arash Ziapour; Reyhane Naderlou; Raziyeh Teimouri; Parisa Janjani; Komali Yenneti: Conceived and designed the experiments; Performed the experiments; Analyzed and interpreted the data; Contributed reagents, materials, analysis tools or data; Wrote the paper.

## Funding statement

This research did not receive any specific grant from funding agencies in the public, commercial, or not-for-profit sectors.

## Data availability statement

Data will be made available on request.

## Declaration of interests statement

The authors declare no conflict of interest.

## Additional information

No additional information is available for this paper.
